# Effective connectivity among the working memory regions during preparation for and during performance of the n-back task

**DOI:** 10.3389/fnhum.2014.00593

**Published:** 2014-08-05

**Authors:** Anna Manelis, Lynne M. Reder

**Affiliations:** ^1^Department of Psychiatry, University of Pittsburgh Medical Center, Western Psychiatric Institute and Clinic, University of PittsburghPittsburgh, PA, USA; ^2^Department of Psychology, Carnegie Mellon UniversityPittsburgh, PA, USA

**Keywords:** working memory, task preparation, fMRI, working memory network, connectivity, graph modeling, IMaGES, LOFS

## Abstract

Recent neuroimaging studies have shown that working memory (WM) task difficulty can be decoded from patterns of brain activation in the WM network during preparation to perform those tasks. The inter-regional connectivity among the WM regions during task preparation has not yet been investigated. We examined this question using the graph modeling methods IMaGES and LOFS, applied to the previously published fMRI data of Manelis and Reder (2013). In that study, subjects performed 1-, 2-, and 3-back tasks. Each block of n-back was preceded by a preparation period and followed by a rest period. The analyses of task-related brain activity identified a network of 18 regions that increased in activation from 1- to 3-back (*Increase* network) and a network of 17 regions that decreased in activation from 1- to 3-back (*Decrease* network). The graph analyses revealed two types of connectivity sub-networks within the *Increase* and *Decrease* networks: “default” and “preparation-related.” The “default” connectivity was present not only during task performance, but also during task preparation and during rest. We propose that this sub-network may serve as a core system that allows one to quickly activate cognitive, perceptual and motor systems in response to the relevant stimuli. The “preparation-related” connectivity was present during task preparation and task performance, but not at rest, and depended on the n-back condition. The role of this sub-network may be to pre-activate a connectivity “road map” in order to establish a top-down and bottom-up regulation of attention prior to performance on WM tasks.

## Introduction

Working memory (WM) is a system involved in on-line maintenance and manipulation of information (Baddeley and Hitch, 1974; Baddeley, 2010). It has a limited capacity (Cowan, 2001; Baddeley, 2010) and is critically important for learning, reasoning and decision-making (Mishkin and Manning, 1978; Petrides and Milner, 1982; Curtis and D'Esposito, 2003; Müller and Knight, 2006). Extensive neuroimaging research demonstrated that when a WM task becomes more difficult, frontal, parietal and striatal regions increase in activation (see Owen et al., 2005; Rottschy et al., 2012, for reviews), while frontal medial and posterior cingulate cortices decrease in activation (e.g., McKiernan et al., 2003; Esposito et al., 2006; Mayer et al., 2010; Manelis and Reder, 2013). Several studies have shown that inter-regional connectivity among the regions in the WM network also changes as a function of WM load (Honey et al., 2002; Wendelken et al., 2008; Kim et al., 2012; Ma et al., 2012; Dima et al., 2014). For example, increases in WM load were characterized by increases in connectivity among the frontal regions (right and left middle frontal gyrus (MFG), and left inferior frontal gyrus (IFG) and supplementary motor area; Honey et al., 2002), between the left parietal and frontal cortices (Ma et al., 2012), and increased right hemisphere dominance for verbal n-back (Dima et al., 2014), but increased left hemisphere dominance in face-matching n-back (Kim et al., 2012). Recent neuroimaging studies revealed that the modulation of brain activation occurs not only during performance on WM tasks of varying difficulty, but also during preparation to perform these tasks when no maintenance or manipulation requirements were yet imposed (Altamura et al., 2010; Manelis and Reder, 2013). For example, activation in the left inferior frontal gyrus (LIFG), anterior cingulate/paracingulate cortex and the left intraparietal sulcus (LIPS) linearly increased during 1- vs. 2- vs. 3-back task performance, but linearly decreased during preparation to perform the corresponding task (Manelis and Reder, 2013). Using a linear SVM [support vector machine (Vapnik, 1995)] classifier, Manelis and Reder demonstrated that the upcoming n-back condition (1- vs. 2- vs. 3-back) could be accurately decoded from the patterns of brain activation recorded in the WM network during preparation periods preceding 1-, 2-, or 3-back tasks. Not only did subjects with higher classification accuracies have more distinct neural representations for each upcoming difficulty level (a reason for why their classification accuracies were higher) they also displayed faster response times (RT) overall and smaller differences between 1-back and 3-back conditions. These results suggest that formation of more distinct neural representations during preparation periods helps subjects to be more efficient during task performance.

If the different preparation periods can be distinguished based on the activation patterns within the WM network, it is also possible that they can be distinguished based on the connectivity within that network. The inter-regional connectivity among the WM regions during task preparation has not yet been investigated in WM studies. In this study, we tested two alternative hypotheses: (1) Given that no information maintenance or manipulation is required during any condition's preparation periods, the connectivity among the WM regions during these preparation periods will resemble the connectivity at rest; vs. (2) The connectivity among the WM regions during preparation periods will resemble the connectivity during task performance. Formation of task-related connectivity before task performance (that is, during task preparation) may be beneficial because such advanced formation may (1) free some neural resources during task performance, and (2) help to activate and transfer the task-related rules and intentions from task preparation to the task itself.

The goal of this paper is to examine effective connectivity within the WM network during WM task preparation, WM task performance and during the rest periods that separate the blocks of the WM tasks. Many effective connectivity algorithms have been used to identify connectivity among regions within a network. Many of these approaches use a confirmatory method that require *a priori* model specification (e.g., DCM), which makes an exhaustive model search across large network of regions almost impossible (Hanson et al., 2007). Another limitation of many effective connectivity approaches is that they were not specifically designed for a multi-subject fMRI data processing and may produce false statistical dependencies by directly combining time series across subjects. A recently developed graphical analysis using the Independent Multiple sample Greedy Equivalence Search (IMaGES) algorithm (Ramsey et al., 2010) used in combination with the Linear non-gaussian Orientation, Fixed Structure (LOFS) algorithm (Ramsey et al., 2011) overcomes these limitations. Because both algorithms were specifically designed to estimate causal relationships based on simultaneous processing of multiple time series of multiple subjects, they do not produce artifacts related to concatenating multiple time series for the analyses (Ramsey et al., 2011). In simulation studies, the combined IMaGES + LOFS method showed very accurate performance on simulated data (Ramsey et al., 2011), while other effective connectivity algorithms had trouble with identification of both inter-regional connections and directions of the identified connections (Smith et al., 2011). For example, on simulated data that consisted of 50 variables used in Smith et al. (2011), IMaGES discovered over 95% of the connections (see Section Effective connectivity analyses for detailed description of how connections were discovered) and LOFS correctly oriented over 80% of these connections (Ramsey et al., 2011).

Based on the fact that using the combination of IMaGES and LOFS produced the most accurate performance among all effective connectivity algorithms in simulation studies, that IMaGES and LOFS were specifically designed to deal with the multi-subject fMRI data and that these methods were successfully used to examine effective connectivity within the language (Boukrina and Graves, 2013; Boukrina et al., 2014) and social brain (Hanson et al., 2013) networks, we decided to use these algorithms in our study. In this paper, we applied IMaGES (to search for connections) and LOFS (to orient the connections discovered by IMaGES) algorithms on Manelis and Reder's (2013) fMRI data to examine effective connectivity within the WM network of regions that increased in activation from 1- to 3-back (*Increase* network) and within the WM network of regions that decreased in activation from 1- to 3-back (*Decrease* network). The connectivity among 18 regions comprising the *Increase* network and, separately, among 17 regions comprising the *Decrease* network was examined during the 1-, 2-, and 3-back tasks, during preparation for these tasks and during rest periods separating the n-back blocks. We focused on identifying two distinct groups of connections—those that were (1) common for all preparation periods, all n-back blocks and rest periods (“default connections”), and those that were (2) common for task preparation and the corresponding n-back conditions, but were not observed during rest periods (“preparation-related connections”).

## Materials and methods

### Subjects and task design

The details pertaining to the study participants, the n-back task and the fMRI methods were published in Manelis and Reder (2013). In short, the dataset consisted of 16 subjects (mean age = 24, 11 females), all of whom were right-handed, native speakers of English, with normal or corrected-to-normal vision. They were treated in accordance with the Carnegie Mellon University Institutional Review Board guidelines.

The subjects were scanned while doing the n-back task at the three levels of difficulty (1-, 2-, and 3-back) with words selected from the MRC Psycholinguistic Database as stimuli. The words were between 4 and 7 letters long and were repeated within, but not between, the blocks. The words were separated with a jittered interval of 2–8 s. The 12-trial blocks of 1-, 2-, and 3-back (10 blocks in each condition) were presented in random order. The duration of a trial was limited to 4 s. Each block was preceded by an 8-s instruction screen, which informed subjects about whether the upcoming block would be 1-, 2-, or 3-back by displaying “1-back,” “2-back,” or “3-back.” A 10–12-s rest period followed each block.

### fMRI acquisition, preprocessing, and GLM analyses

As described in Manelis and Reder (2013), the fMRI data were collected using a Siemens 3T Verio MR system. We acquired a high-resolution structural image (0.8 × 0.8 × 0.8 mm) using MPRAGE (*TR* = 1800 ms, *TE* = 2.22 ms, FOV = 205, *FA* = 9°, number of slices = 256), functional data using a gradient-echo echo-planar sequence (*TR* = 2000 ms, *TE* = 30 ms, FOV = 205, *FA* = 79°, 36 slices, 3.2 × 3.2 × 3.2 mm), and field maps with the same resolution as the BOLD images using a gradient-echo sequence (*TR* = 394 ms, *FA* = 60°, *TE* = 5.1 and 7.56 ms).

The fMRI data were preprocessed and analyzed using FSL 4.1.7 (www.fmrib.ox.ac.uk/fsl). Preprocessing included non-linear noise reduction performed using SUSAN (http://fsl.fmrib.ox.ac.uk/fsl/fslwiki/SUSAN), motion correction with MCFLIRT (Jenkinson et al., 2002), fieldmap-based EPI unwarping using PRELUDE+FUGUE (Jenkinson, 2003), non-brain removal using BET (Smith, 2002), spatial smoothing using a Gaussian kernel of FWHM 6 mm, grand-mean intensity normalization of the entire 4D dataset by a single multiplicative factor, high-pass temporal filtering (Gaussian-weighted least-squares straight line fitting, with sigma = 50.0 s). The Probabilistic Independent Component Analysis (ICA; Beckmann and Smith, 2004), implemented using FSL's Multivariate Exploratory Linear Decomposition into Independent Components (http://www.fmrib.ox.ac.uk/fsl/melodic/index.html), served to identify “noise” components (Tohka et al., 2008; Kelly et al., 2010) that were then removed using the fsl_regfilt script.

The preprocessed data were used in the GLM analysis with 6 regressors (1-, 2-, and 3-back instruction periods, and 1-, 2-, and 3-back task performance blocks) that examined two linear trends in brain activation: 1-back<2-back<3-back and 1-back>2-back>3-back. Co-registration of BOLD images with the MNI152_T1_2 mm template was carried out using FLIRT (Jenkinson and Smith, 2001; Jenkinson et al., 2002). As described in Manelis and Reder (2013), a group analysis was conducted using the Randomise v2.1 tool (http://www.fmrib.ox.ac.uk/fsl/randomise/index.html) with the whole brain as a mask, 5 mm smoothing, 5000 permutations and correction for multiple comparisons at the voxel-wise FWE-controlled threshold *p* < 0.05). This analysis revealed a network of 18 regions that linearly increased activation from 1- to 3-back (*Increase* network; see Table 1) and a network of 17 regions that linearly decreased activation from 1- to 3-back (*Decrease* network; see Table 1) that were used in the effective connectivity analyses described below.

**Table 1:** **Parametric changes in brain activation as a function of working memory load during task performance**.

	**Region**	**Abbreviation**	**n-voxels**	**Z-max**	***x***	***y***	***z***
**PARAMETRIC INCREASES IN ACTIVATION (1-back<2-back<3-back) DURING TASK PERFORMANCE**							
L	Anterior cingulate c./paracingulate g.	ACC	818	9.99	−2	22	42
R	Inferior parietal s.	RIPS	503	8.29	40	−46	44
L	Orbitofrontal c.	LOFc	447	9.66	−32	24	−6
L	Inferior parietal s.	LIPS	407	8.0	−34	−52	40
L	Superior frontal g.	LSFG	388	7.4	−28	8	62
R	Frontal pole	RFP	370	8.22	42	48	22
R	Precuneus	Rprecun	314	7.99	2	−62	48
L	Frontal pole	LFP	313	7.58	−38	56	10
L	Inferior frontal g.	LIFG	300	8.21	−40	8	26
L	Middle frontal g.	LMFG	277	7.66	−48	36	22
R	Superior frontal g.	RSFG	262	6.87	28	12	60
R	Insular c.	Rins	177	8.88	34	24	−2
L	Basal ganglia	Lbas	160	7.2	−16	0	14
R	Lateral occipital c., superior	RLOCs	97	7.1	32	−66	46
L	Thalamus	Lthal	52	6.49	−8	−18	8
R	Cerebellum	Rcereb	27	5.83	36	−70	−28
L	Lateral occipital c., superior	LLOCs	26	6.42	−16	−74	52
R	Middle frontal g.	RMFG	10	5.92	44	34	38
**PARAMETRIC DECREASES IN ACTIVATION (3-back<2-back<1-back) DURING TASK PERFORMANCE**							
L	Planum polare	LPlanPol	1599	10.4	−42	−16	−8
R	Parietal operculum	RParOperc	1305	8.67	48	−30	22
L	Anterior cingulate/Medial frontal c.	LFmed	968	8.22	−2	54	−4
B	Juxtapositional lobule c.	BJuxt	916	9.0	2	−8	48
L	Posterior cingulate g., posterior	LPCCp	591	8.29	−10	−50	28
R	Right pre−central g.	RPrec	312	7.1	36	−18	48
R	Planum polare	RPlanPol	228	7.14	42	0	−18
L	Posterior cingulate g., anterior	LPCCa	200	8.47	−14	−30	38
L	Post−central/pre−central g.	LPostPrec	64	8.2	−36	−18	40
R	Lateral occipital c., inferior	RLOCi	60	6.52	54	−70	8
R	Post−central g., anterior	RPosta	31	6.41	56	−16	48
R	Temporal pole	RTP	17	6.55	40	24	−26
B	Subcallosal c.	BSub	13	5.9	0	16	−12
R	Frontal pole	RFP	13	5.85	36	36	−16
R	Post−central g., middle part	RPostm	12	6	30	−32	70
L	Occipital pole	LOccipP	11	5.69	−26	−94	24
R	Post−central g., posterior	RPostp	10	5.71	26	−36	56

The images were thresholded at voxel-wise FWE-corrected p < 0.05. The abbreviations for all regions are in the third column. L, left; R, right; B, bilateral; g., gyrus; s., sulcus; c., cortex.

### Effective connectivity analyses

The effective connectivity analyses were conducted using graphical causal modeling using IMaGES (the Independent Multiple sample Greedy Equivalence Search) and LOFS (Linear non-gaussian Orientation, Fixed Structure) algorithms (Ramsey et al., 2010, 2011, 2014; Mumford and Ramsey, 2014) implemented using the TETRAD IV (version 5.0.0-1; http://www.phil.cmu.edu/projects/tetrad) software. Separate connectivity analyses were conducted for the regions within the *Increase* network and the regions within the *Decrease* network. First, we extracted the time series from each of the ROIs identified in the Manelis and Reder (2013) study for each subject. All those ROIs have already been corrected for multiple comparisons in the original GLM that identified those ROIs. Each of these ROIs is a node in the network whose connectivity we examine in the analyses described below. Each of the three n-back conditions was associated with 10 preparation periods lasting for 4 TRs each, which provided us with a total of 40 data points per preparation condition per subject per ROI. The length of each block of n-back was at least 21 TRs (but could be longer given the self-paced nature of the task), which provided us with at least 210 data points per n-back condition per subject per ROI. There were 30 rest periods in the experiment. The length of each rest periods was 5–6 TRs. Only the last 3–4 TRs were included in the data analyses, which provided us with at least 90 data points per subject per ROI (3 TRs × 30 rest periods).

Second, the extracted time series combined across subjects and ROIs were used as input to the IMaGES algorithm with increasing penalty discounts (Ramsey et al., 2010). IMaGES is a Bayesian search algorithm that produces a Markov equivalence class of models that have the same structure (the same connections between the nodes without considering the direction of those connections). For each set of regions, IMaGES starts with an empty graph. It tests all possible models with one connection and selects one model with the highest Bayesian Information Criterion (BIC) score averaged across several datasets. The algorithm continues to add connections between nodes until further connections do not improve the BIC score. At that point, the process is reversed, removing connections from the model, one at a time until the BIC score can no longer be improved. In some cases, Greedy Equivalence Search can produce the graphs where the three nodes (e.g., three brain regions) are connected to each other (i.e., “triangulation”). Given that we are trying to estimate causal relationships among latent variables, such “triangulation” can lead to false conclusions (Ramsey et al., 2010). “Triangulation” and the inherent possibility of spurious causal connections can be avoided by increasing the penalty function in the BIC score (Ramsey et al., 2010). In this study, we used the option “find first non-triangular” to search for a graph that does not contain “triangulation” [directed acyclic graph (DAG)].

Third, after IMaGES identified a DAG for the set of regions, the DAG is fed to the LOFS algorithm (Ramsey et al., 2011). LOFS determines the orientation (direction) of each connection by exploiting the fact that the residuals of any incorrect linear model will be more Gaussian than the residuals of the correct model with independent non-Gaussian sources of error (Ramsey et al., 2011). In our study, the degree of non-Gaussianity was estimated using the Anderson–Darling score (Anderson and Darling, 1952). After LOFS oriented the connection, each graph consisted of nodes and arrows (or edges, or connections) that connect some of those nodes thus depicting causal relationships between them. For example, the arrow in the LMFG→LIFG expression shows that the changes in LMFG activation influence the changes in LIFG activation. Finally, after the connections were detected and oriented, the outcome of the LOFS algorithm is submitted to a SEM (structural equation modeling) estimator that estimates model goodness of fit to each set of data by estimating the values of parameters for a SEM parametric model with a regression optimizer. Accurate regression estimates presuppose that the input parametric model be a DAG, and its associated statistics are based on a linear, Gaussian model.

The effective connectivity within *Increase* and *Decrease* networks was assessed for each of the three n-back conditions, for each of the three corresponding preparation periods and also at rest. The rest periods were analyzed starting 4 s after the end of a preceding n-back block. The effective connectivity for the seven conditions (rest, 1, 2, 3-back preparation periods and 1, 2, 3-back task performance periods) were compared to identify the connections that were common for all these conditions and those that were common for the corresponding preparation and n-back conditions but were absent at rest.

## Results

### Behavioral and neuroimaging activation analyses

**Figure 1:**
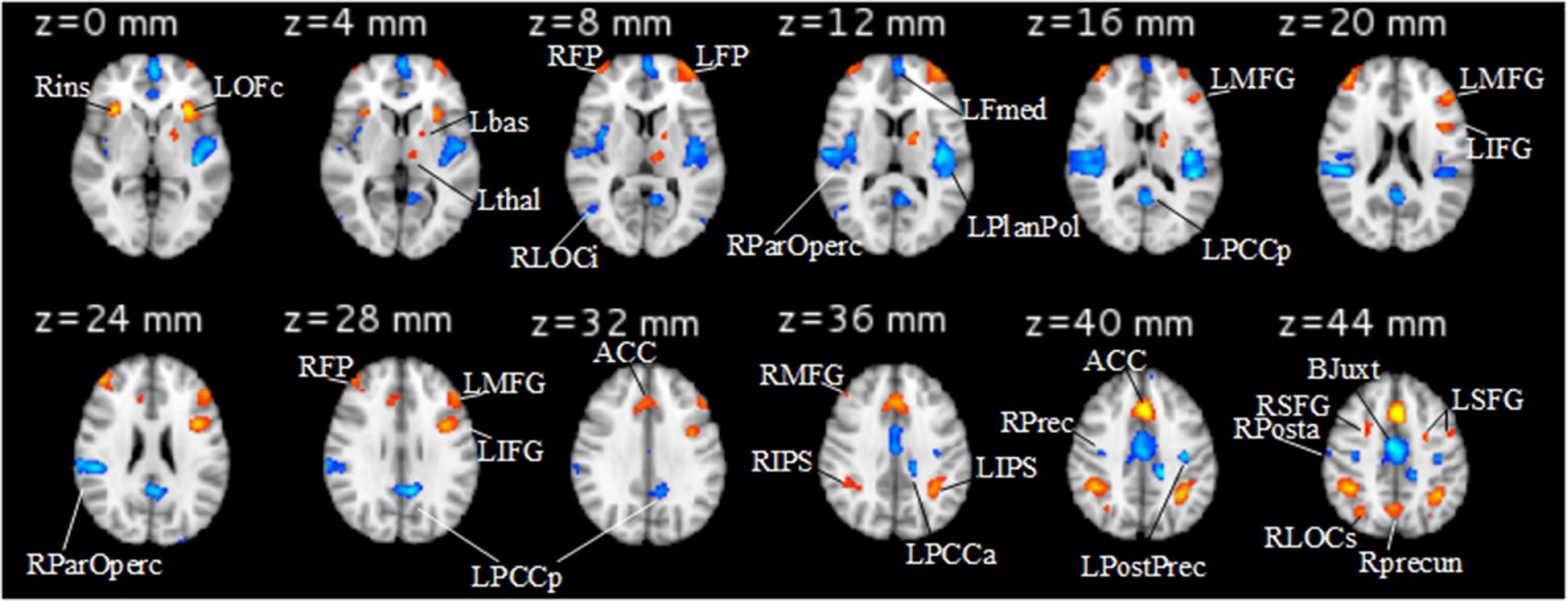
***Increase* (1-back<2-back<3-back, shown in red) and *Decrease* (1-back>2-back>3-back, shown in blue) networks identified in the GLM analysis**. The abbreviations for the brain regions are described in Table 1. Left hemisphere is on the right and right hemisphere is on the left.

The details of the behavioral analysis and the univariate analysis of neuroimaging data are described in Manelis and Reder (2013). Consistent with previous work (e.g., Braver et al., 1997; Nystrom et al., 2000), they found that, as the n-back task difficulty increased, performance slowed [*F*_(2, 30)_ = 46.4, *p* < 0.001] and became less accurate [*F*_(2, 30)_ = 22.5, *p* < 0.001]. Table 1 and Figure 1 report regions (and their corresponding abbreviations) that increased in activation as the task difficulty increased, and also those regions that decreased in activation as the task difficulty increased.

### Effective connectivity analysis

**Figure 2:**
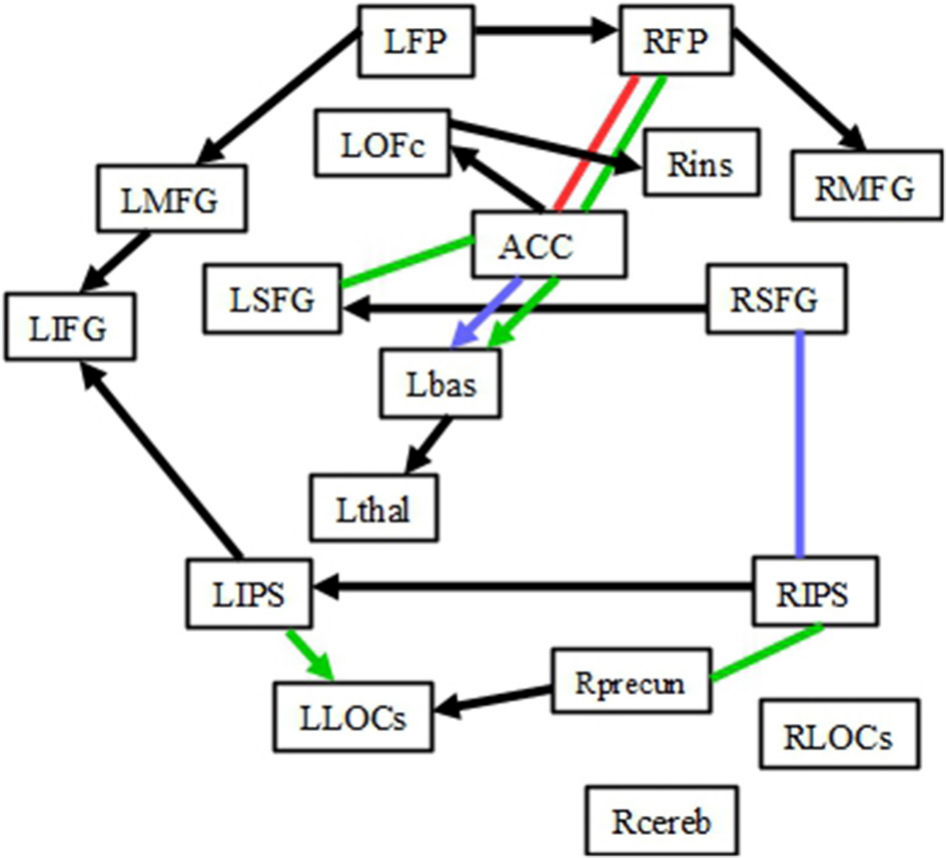
**Connectivity within the *Increase* network**. Black lines depict the connections common for 1-, 2-, 3-back preparation, 1-, 2-, 3-back task performance and also rest periods. The red line represents the connection common for 1-back preparation and task, but not rest periods. Blue lines represent the connections common for 2-back preparation and task, but not rest periods. Green lines represent the connections common for 3-back preparation and task, but not rest periods. Please note that the direction of each arrow represents the directionality for the majority of connections (not all of them). The lines without arrows represent the connections whose directions differed for preparation and task performance conditions.

**Figure 3:**
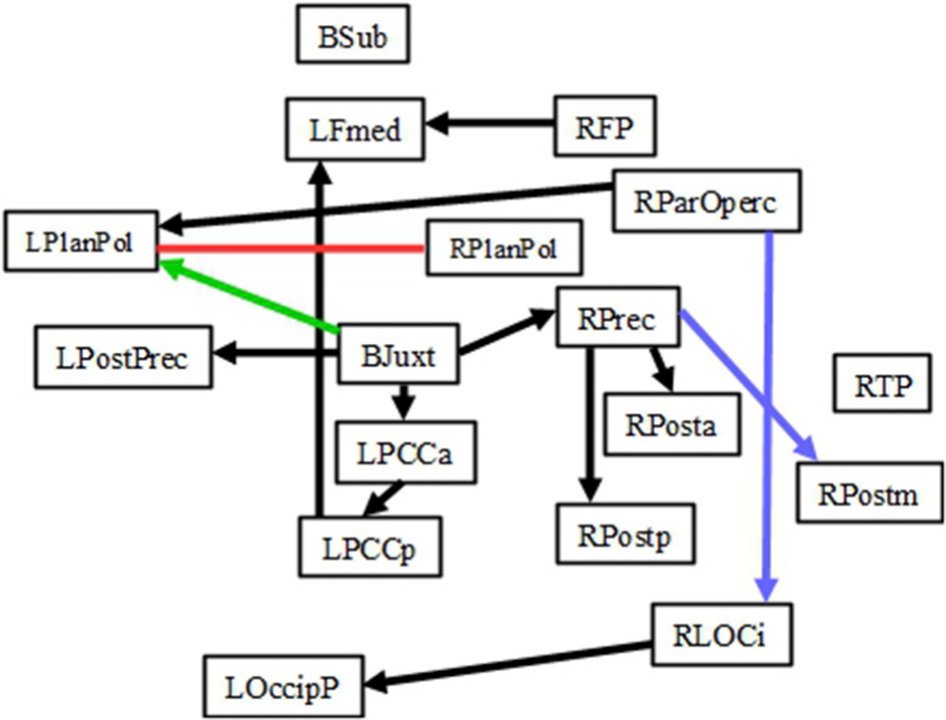
**Connectivity within the *Decrease* network**. Black lines depict the connections common for 1-, 2-, 3-back preparation, 1-, 2-, 3-back task performance and also rest periods. The red line represents the connection common for 1-back preparation and task, but not rest, periods. Blue lines represent the connections common for 2-back preparation and task, but not rest, periods. The green line represents the connection common for 3-back preparation and task, but not rest, periods. Please note that the direction of each arrow represents the directionality for the majority of connections (not all of them). The lines without arrows represent the connections whose directions differed for preparation and task performance conditions.

The results of IMaGES and LOFS analyses are presented in Tables 2 (for *Increase* network) and 3 (for *Decrease* network). The tables report mean and standard deviations of SEM coefficients across subjects. The connections between pairs of regions are in the left column. The directions of the connections are shown with the directed arrows “→.” For example, the sentence A→B means that changes in activation in the region A cause changes in activation in the region B. Sometimes, IMaGES would find that a certain connection existed in several conditions; however, LOFS determined that the direction of this connectivity varied among the specific conditions. In cases such as these (i.e., when the direction of the connection was different from that shown in the left column of Tables 2, 3), we placed the arrow “←” left from the SEM coefficient. Although Tables 2, 3 report all connections and their directions identified by IMaGES and LOFS, the discussion will focus only on those connections that were common for all seven conditions and on the connections that were common for the corresponding task preparation and task performance periods (e.g., 1-back preparation and 1-back performance) but were absent at rest. Figure 2 (*Increase* network) and Figure 3 (*Decrease* network) also focus only on these two sets of the findings.

**Table 2:** **Inter-regional connectivity within the *Increase* network**.

**Connections**	**Intro1**	**1-back**	**Intro2**	**2-back**	**Intro3**	**3-back**	**Rest**
RFP→RMFG	0.72 (0.3)	0.94 (0.3)	← 0.59 (0.2)	0.96 (0.3)	← 0.22 (0.2)	0.97 (0.3)	← 0.21 (0.1)
LIPS→LIFG	← 0.28 (0.2)	0.42 (0.2)	← 0.45 (0.2)	0.52 (0.2)	0.7 (0.3)	0.34 (0.2)	0.32 (0.3)
LMFG→LIFG	0.45 (0.2)	0.4 (0.2)	0.69 (0.2)	← 0.51 (0.3)	← 1.08 (0.4)	0.31 (0.1)	0.34 (0.1)
LFP→RFP	0.43 (0.3)	← 0.82 (0.2)	← 0.81 (0.2)	← 0.82 (0.2)	0.34 (0.2)	0.49 (0.1)	0.36 (0.2)
LFP→LMFG	← 0.93 (0.2)	0.69 (0.2)	0.33 (0.2)	0.26 (0.2)	← 0.89 (0.2)	0.38 (0.2)	0.41 (0.2)
ACC→LOFc	← 0.89 (0.2)	0.76 (0.2)	0.51 (0.2)	0.77 (0.2)	0.78 (0.2)	← 0.86 (0.2)	0.43 (0.2)
LOFc→Rins	0.86 (0.2)	0.74 (0.1)	← 0.41 (0.2)	0.76 (0.1)	0.66 (0.2)	0.78 (0.1)	← 0.47 (0.1)
Lbas→Lthal	1.07 (0.3)	1.08 (0.3)	← 0.33 (0.2)	0.97 (0.2)	1.03 (0.4)	← 0.29 (0.1)	← 0.48 (0.2)
Rprecun→LLOCs	← 0.59 (0.3)	0.68 (0.2)	0.75 (0.3)	0.71 (0.2)	0.5 (0.3)	← 0.61 (0.2)	0.70 (0.2)
RIPS→LIPS	0.29 (0.2)	← 0.61 (0.2)	0.51 (0.1)	0.82 (0.2)	0.78 (0.2)	0.65 (0.1)	0.81 (0.2)
RSFG→LSFG	0.74 (0.2)	0.4 (0.2)	← 0.91 (0.2)	0.5 (0.2)	0.67 (0.2)	← 0.61 (0.1)	← 0.85 (0.1)
LIFG→ACC	0.33 (0.3)	0.61 (0.2)		0.39 (0.2)	0.39 (0.2)	0.24 (0.1)	0.19 (0.2)
LSFG→LMFG	0.99 (0.4)	← 0.27 (0.1)	0.66 (0.3)	0.34 (0.2)		0.62 (0.1)	0.6 (0.2)
RIPS→RLOCs		0.94 (0.2)	0.96 (0.3)	0.96 (0.3)	0.78 (0.3)	0.92 (0.2)	0.73 (0.3)
RIPS→RFP	0.28 (0.3)	← 0.24 (0.1)			0.39 (0.2)	← 0.64 (0.2)	0.22 (0.2)
LIPS-LSFG	← 0.17 (0.2)	0.17 (0.1)		0.39 (0.2)			0.74 (0.2)
RSFG→RFP	0.32 (0.3)					← 0.33 (0.2)	0.21 (0.2)
LLOCs→RLOCs					0.25 (0.2)	← 0.39 (0.3)	0.27 (0.2)
RSFG→ACC							0.75 (0.2)
RFP→ACC	**← 0.00 (0.3)**	**0.34 (0.1)**			**0.28 (0.1)**	**← 0.5 (0.2)**	
RSFG→RIPS		0.18 (0.2)	**0.69 (0.2)**	**← 0.85 (0.2)**	0.63 (0.4)		
ACC→Lbas	0.66 (0.1)		**0.37 (0.2)**	**0.59 (0.2)**	**0.66 (0.3)**	**0.38 (0.2)**	
LSFG→ACC			0.9 (0.3)		**0.35 (0.2)**	**← 0.76 (0.2)**	
LIPS→LLOCs		← 0.46 (0.1)			**0.55 (0.5)**	**0.82 (0.4)**	
RIPS→Rprecun				1.19 (0.3)	**← 0.17 (0.2)**	**0.48 (0.3)**	
Rcereb→Rprecun			0.21 (0.3)			0.13 (0.4)	
RLOCs→Rprecun	0.56 (0.4)		0.86 (0.4)				
LFP→LIPS						0.16 (0.1)	
LFP→Rprecun		0.82 (0.3)					
LMFG→RMFG	0.34 (0.3)						
Rcereb→LIPS		0.13 (0.1)					
RIPS→Rins					0.19 (0.2)		
RLOCs→LIPS	0.16 (0.1)						
RMFG→LSFG					0.07 (0.2)		
Rprecun→Rcereb				0.74 (0.3)			
Rprecun→RFP	0.08 (0.2)						

The arrow “←” shows the connections whose direction was opposite to one described in the left column titled “Connections.” Standard deviations of mean SEM coefficients across the subjects are in parentheses. The connections formed during the preparation periods and carried over to the corresponding task performance periods, but absent during rest, are shown in bold.

**Table 3:** **Inter-regional connectivity within the *Decrease* network**.

**Connections**	**Intro1**	**1-back**	**Intro2**	**2-back**	**Intro3**	**3-back**	**Rest**
LPCCa→LPCCp	0.59 (0.4)	1.1 (0.3	← 0.17 (0.1)	0.55 (0.3)	0.83 (0.5)	1.13 (0.2)	1.04 (0.3)
LPCCp→LFmed	← 0.48 (0.4)	0.64 (0.2)	0.52 (0.3)	← 0.54 (0.2)	0.5 (0.3)	0.73 (0.2)	0.73 (0.1)
RFP→LFmed	← 0.59 (0.2)	0.25 (0.2)	0.35 (0.3)	0.73 (0.2)	0.42 (0.4)	← 0.58 (0.2)	← 0.48 (0.2)
BJuxt→LPCCa	← 1.3 (0.4)	← 0.82 (0.2)	0.52 (0.1)	0.63 (0.1)	0.61 (0.1)	0.63 (0.1)	0.55 (0.1)
BJuxt→LPostPrec	0.61 (0.2)	0.6 (0.2)	0.61 (0.2)	0.59 (0.2)	← 1.06 (0.3)	0.62 (0.2)	← 0.55 (0.3)
BJuxt→RPrec	0.73 (0.2)	← 0.39 (0.1)	0.74 (0.3)	0.7 (0.2)	0.26 (0.2)	0.69 (0.2)	0.37 (0.2)
RLOCi→LOccipP	0.69 (0.2)	0.71 (0.3)	0.7 (0.3)	0.69 (0.2)	0.64 (0.4)	← 0.31 (0.2)	0.67 (0.2)
RParOperc→LPlanPol	← 0.72 (0.2)	0.63 (0.2)	← 1.04 (0.2)	← 0.67 (0.1)	0.47 (0.2)	0.51 (0.1)	0.74 (0.1)
RPrec→RPosta	0.74 (0.3)	0.77 (0.2)	0.78 (0.2)	0.76 (0.2)	← 0.35 (0.2)	0.76 (0.2)	0.71 (0.2)
RPrec→RPostp	0.84 (0.2)	0.46 (0.3)	0.81 (0.2)	0.72 (0.2)	← 0.38 (0.2)	0.76 (0.2)	← 0.46 (0.1)
BJuxt→RParOperc	0.31 (0.2)	0.76 (0.1)		0.32 (0.1)			← 0.52 (0.1)
RPostp→RPostm	1.17 (0.3)	← 0.31 (0.2)			1.1 (0.4)		1.09 (0.4)
RParOperc→RPlanPol				0.86 (0.1)		0.86 (0.2)	← 0.55 (0.2)
RTP→RPlanPol	0.18 (0.2)				0.34 (0.2)		← 0.72 (0.5)
LPCCa→RLOCi					0.95 (0.4)		0.80 (0.2)
RPostp→LPCCa							0.1 (0.09)
RParOperc→RLOCi	0.64 (0.3)		**0.58 (0.4)**	**0.64 (0.2)**		0.48 (0.2)	
RPlanPol→LPlanPola	**← 0.9 (0.2)**	**0.14 (0.1)**	0.29 (0.2)		0.15 (0.1)		
BJuxt→LPlanPol			0.47 (0.1)		**0.24 (0.1)**	**0.29 (0.1)**	
RPrec→RPostm			**1.12 (0.3)**	**1.02 (0.4)**		1.05 (0.4)	
RTP→LFmed		0.11 (0.1)				0.17 (0.2)	
RFP→RTP			0.85 (0.6)				
BJuxt→RTP				0.7 (0.5)			
RLOCi→BJuxt		0.06 (0.1)					

The arrow “←” shows the connections whose direction was opposite to one described in the left column titled “Connections.” Standard deviations of mean SEM coefficients across the subjects are in parentheses. The connections formed during the preparation periods and carried over to the corresponding task performance periods, but absent during rest, are shown in bold.

#### Connections common across all conditions in the experiment including rest: Increase network

Within the *Increase* network, 11 connections were present during rest, all three preparatory periods and all three task performance conditions. While the direction of those connections varied depending on the condition, in general, those connections formed a network linking frontal and parietal regions as well as the right and left hemispheres: RMFG-RFP, RFP-LFP, LFP-LMFG, LMFG-LIFG, LIFG-LIPS, LIPS-RIPS, Rins-LOFc, LOFc-ACC, Lbas-Lthal, LLOCs-Rprecun, RSFG-LSFG (black arrows in Figure 2). Given the verbal nature of the task, it is not surprising that there were more left-sided connections than right-sided ones.

#### Connections that existed during preparation periods and corresponding task conditions, but were absent at rest: Increase network

The graph modeling analysis revealed that some connections were present during corresponding preparation and task periods, but not at rest (colored arrows and lines in Figure 2). The number of common connections increased for more difficult tasks. One common connection (RFP-ACC) was found for 1-back preparation and 1-back task performance. Two common connections (RSFG-RIPS and ACC-Lbas) were found for 2-back preparation and 2-back task performance. Five common connections (RFP-ACC, ACC-Lbas, ACC-LSFG, LIPS-LLOCs, and RIPS-Rprecun) were found for 3-back preparation and 3-back task performance periods.

#### Connections common across all conditions in the experiment including rest: Decrease network

Within the *Decrease* network, 10 connections (BJuxt-RPrec, Rprec-RPosta, Rprec-Rpostp, BJuxt-LPostPrec, BJuxt-LPCCa, LPCCa-LPCCp, LPCCp-LFmed, RFP-LFmed, RParOperc-LPlanPol, RLOCi-LOccip) existed throughout the experiment during rest, all preparatory and all task performance conditions (Table 3, Figure 3). Those connections linked the right and left hemispheres (e.g., RParOperc-LPlanPol, RLOCi-LOccip) as well as the frontal, parietal and occipital regions, often through the structures located along the midline (e.g., BJuxt-RPrec, BJuxt-LPostPrec, BJuxt-LPCCa, LPCCa-LPCCp, LPCCp-LFmed). BJuxt was a point of origin for several connections as the changes in activation in this region often caused changes in activation in LpostPrec, LPCCa, and RPrec. RPrec, in turn, often caused changes in activation in RPosta and RPostp. In contrast, the LFmed was a point of convergence as its activation was often caused by changes in activation in RFP and LPPCp.

#### Connections that existed during preparation periods and corresponding task conditions, but were absent at rest: Decrease network

In the *Decrease* network, there was a common LPlanPolar-RPlanPolar connection for 1-back preparation and 1-back task performance. In the 2-back condition, there were common RPrec→RPostm and RparOperc→RLOCi connections. In the 3-back condition, there was a common BJuxt→LPlanPolar connection.

#### Connections observed during preparation periods and at rest, but absent during task performance

In the Increase network, there were no connections that were common for the preparation periods and rest that were not also present during task performance. In the Decrease network, RTP-RPlanPol connectivity was found during preparation for the 1- and 3-back tasks and also at rest, but not during n-back task performance. In the preparation conditions, activation in RTP caused activation in RPlanPol, while at rest, activation in RPlanPol caused activation in RTP. LPCCa→RLOCi connectivity was observed during preparation for the 3-back task and at rest but not during other conditions.

## Discussion

This is the first study to examine effective connectivity within the WM network during preparation to perform the n-back task, during performance on these tasks, and during rest periods that separate the n-back blocks. We applied recently developed graph modeling methods IMaGES and LOFS to Manelis and Reder's (2013) fMRI data in order to examine connectivity within the *Increase* and *Decrease* networks. The regions within the *Increase* network increased in activation with an increase in WM load and were consistent with those previously reported in other WM studies (see Owen et al., 2005, for a review). The regions within the *Decrease* network decreased in activation when WM load or attentional demands increased (e.g., McKiernan et al., 2003; Esposito et al., 2006; Mayer et al., 2010). These included the regions associated with the default mode network (e.g., Raichle et al., 2001; Greicius et al., 2003; Buckner et al., 2008). The results of the graph analyses revealed two types of connectivity sub-networks within the *Increase* and *Decrease* networks: “default” and “preparation-related.”

### “Default” sub-network within the increase and decrease networks

We called one sub-network “default” connectivity because those connections existed regardless of whether a subject was preparing for the task, was performing the task or was at rest. “Default” connections were found in both *Increase* (RMFG-RFP, RFP-LFP, LFP-LMFG, LMFG-LIFG, LIFG-LIPS, LIPS-RIPS, Rins-LOFc, LOFc-ACC, Lbas-Lthal, LLOCs-Rprecun, RSFG-LSFG) and *Decrease* (BJuxt-RPrec, Rprec-RPosta, Rprec-Rpostp, BJuxt-LPostPrec, BJuxt-LPCCa, LPCCa-LPCCp, LPCCp-LFmed, RFP-LFmed, RParOperc-LPlanPol, RLOCi-LOccip) networks. Those connections were present across all conditions despite the fact that activation in these regions depended on WM load in the task. While it may seem surprising that the patterns of connectivity did not resemble the patterns of activation and did not change in a linear manner, it is possible that stable connectivity is beneficial in those situations in which activity in brain regions is determined by external requirements that unpredictably change during the experiment.

The “default” connections linked 17 of the 18 regions in the *Increase* network and 14 of the 17 regions in the *Decrease* network, thus connecting most of the regions within the WM network to allow integration and quick propagation of incoming information when the task requirements changed. Analogous to the default mode network (e.g., Raichle et al., 2001; Greicius et al., 2003; Buckner et al., 2008), “default” connectivity may play a fundamental role in monitoring the environment, cognitive requirements, and motor responses. PFC and IPS belong to the attentional network (Corbetta, 1998) and are thought to also be involved in task preparation (Brass and von Cramon, 2004). Connectivity among these regions modulates attentional control (Wang et al., 2010), while disconnection among these regions results in impaired attention (Neufang et al., 2011). “Default” connectivity between PFC and IPS regions may help to maintain alertness throughout the experiment thereby enabling responses to stimuli in a timely manner. “Default” connectivity across primary, sensory and motor regions may help individuals to respond “as quickly as possible” when prompted by a task.

Honey et al. (2002) found connectivity between PFC (LIFG) and posterior parietal cortex during the 1- and 2-back conditions. In our study, we found LIFG-LIPS connectivity not only for the 1, 2, and 3-back conditions, but also for all the preparatory and rest conditions. Given the verbal nature of our task and the fact that this connection may be important for mediating articulatory rehearsal (Honey et al., 2002), the presence of this connection in all conditions may have been necessary to facilitate the input of new verbal information during the task. Ma et al. (2012) reported that WM load modulates connectivity within the fronto-parietal network. In contrast to their finding that WM load modulates left parietal→LIFG and right parietal→left parietal connectivity, we found that these connections were present even at rest, and were not modulated by either actual or expected task difficulty. Such differences can be explain by several factors that include differences in the tasks, differences between DCM and IMaGES in modeling effective connectivity and the fact that we examined connectivity not only at task, but also during preparatory and rest periods.

The *Decrease* network includes several regions that belong to the default mode network (e.g., PCC, Fmed). The function of the default mode network is still debated. For example, one group of researchers argues that its activation is related to task-unrelated thoughts (e.g., McKiernan et al., 2006), another that it reflects fundamental functional organization (e.g., Raichle and Snyder, 2007; Vincent et al., 2007), and still others argue that it reflects recent experiences (e.g., Albert et al., 2009; Hasson et al., 2009; Tambini et al., 2010). Connectivity among the regions that decrease in activation during task performance was often examined at rest rather than during task performance; however, some studies have shown that these regions might be equally important for cognitive functioning as those regions that increase in activation during a task (e.g., Sambataro et al., 2010; Yakushev et al., 2013). Our study supports this latter idea by showing that both *Increase* and *Decrease* networks contain “default” connections whose role may be to enable an immediate response to changes in the environment.

### “Preparation-related” sub-network within the increase and decrease networks

Connectivity in the “preparation-related” sub-network was formed during task preparation and carried over to task performance, but not rest periods. The number of such connections depended on task difficulty. Two connections (one in the *Increase* network [RFP-ACC] and one in the *Decrease* network [RPlanPol-LPlanPol]) comprised the “preparation-related” connectivity sub-network during the 1-back task. Four connections (two in the *Increase* network [RSFG-RIPS and ACC-Lbas] and two in the *Decrease* network [RPrec→RPostm and RparOperc→RLOCi]) comprised the “preparation-related” sub-network during the 2-back task. Six connections (five in the *Increase* network [RFP-ACC, ACC-Lbas, ACC-LSFG, LIPS-LLOCs, and RIPS-Rprecun] and one in the *Decrease* network [BJuxt→LPlanPolar]) comprised “preparation-related” sub-network during 3-back.

If the “preparation-related” connectivity within the WM load networks reflect only general task preparation and rule activation, then the number of connections formed during preparation should not depend on the anticipated task difficulty. However, in this study, the number of “preparation-related” connections increased with an increase in the level of expected task difficulty, suggesting that preparation is specific to the level of cognitive demand in the anticipated task. Because different n-back conditions likely require the use of different cognitive strategies, different preparatory conditions were associated with the pre-formation of different connections. Many of those connections involved the ACC, which plays a role in conflict monitoring (e.g., Botvinick et al., 2001, 2004) and in anticipation of conflict monitoring (Sohn et al., 2007). The 1-back condition is the least demanding. It was associated with only a weak (based on the SEM coefficient) ACC→RFP connectivity during preparation and RFP→ACC connectivity at task. Considering that the RFP is associated with time-based prospective memory tasks for both words and pictures (Volle et al., 2011) and with visuospatial prospective memory (Costa et al., 2013), the ACC→RFP connectivity may reflect the regulation processes that change future intentions based on the current state of conflict (e.g., response error) detected by the ACC. The RFP→ACC connectivity, in contrast, may reflect the top-down process that regulates the perception of conflict based on the adjusted prospective goals and intentions.

The 2- and 3-back conditions are more difficult than the 1-back condition and likely involve different strategies to ensure optimal performance. Preparatory processes presumably involve inhibiting the n-back rules specific to the previous n-back block, activating the rules specific for the upcoming block, and establishing some level of cognitive control before a given n-back block starts. Both preparation and task performance in the 2-back and 3-back conditions involved the ACC→Lbas connectivity: the increase in the ACC activation caused the increase in the Lbas activation. The ACC is involved in conflict monitoring (Botvinick et al., 2004). The conflict may arise from the need to use new rules for the upcoming block, from the emotional reaction to the objective or subjective task difficulty and from the perception of response errors. These information may be task-irrelevant and may interfere with task performance. After the ACC detects such information, the activation is spread to the Lbas that inhibits irrelevant information (Yehene et al., 2008). Thus, the ACC→Lbas connectivity may serve for detection and inhibition of irrelevant to the current task information to ensure optimal task performance. During the 3-back task performance, activation in the ACC causes not only a change in Lbas activation but also changes in LSFG and RFP activation, thus spreading activation to frontal regions involved in on-line monitoring and manipulation of information [LSFG (du Boisgueheneuc et al., 2006)] as well as in prospective memory (RFP; e.g., Volle et al., 2011; Costa et al., 2013). In contrast, during preparation periods preceding 3-back trials, activation in the LSFG and RFP caused changes in ACC activation, thus preparing this region for optimal functioning during the demanding 3-back task.

“Preparation-related” connectivity within the parietal cortex (RIPS-Rprecun) and between the parietal and occipital cortices (LIPS-LLOCs) was unique for 3-back preparation and task performance. Together with the “default” RIPS-LIPS and RPrecun-LLOCs connections, they formed a fully connected parieto-occipital network whose function may have been to prepare to integrate and to integrate information about the spatial position of each stimulus in the n-back sequence. This information could then be transferred to the LIFG through the “default” LIPS→LIFG connection for further processing (i.e., evaluation, updating and manipulation) in the frontal cortex. Taken together, the role of the “preparation-related” connectivity sub-network is to establish a top-down and bottom-up regulation of attention prior to performance on a difficult WM task and to pre-activate a connectivity “road map” for subsequent task performance. This early formation of connectivity may be an efficient way to cope with the high processing demands during a task by decreasing the number of connections that have to be formed and allowing more resources to be allocated to the formation of other connections during the task.

### Does the connectivity among the WM regions during preparation periods resembles the connectivity at rest?

Neither the preparation nor the rest periods require maintenance or manipulation of information on-line. As such, it is reasonable to propose that the preparation-related connectivity might resemble the connectivity at rest. To test this hypothesis we examined the connections that were common for task preparation and rest, but were absent during task performance. Surprisingly, we found no such connections in the Increase network. In the Decrease network, we identified two connections common for preparation and rest, but not task performance: One for the 3-back preparation (RTP-RPlanPol and LPCCa→RLOCi) and one such connection for the 1-back preparation (RTP-RPlanPol). The fact that there were more common connections for task preparation and task performance (but not rest) than for task preparation and rest (but not task performance) suggests that the connectivity during task preparation is more similar to that during task performance than to the connectivity at rest. These results also suggest that, despite the fact that the task preparation periods do not involve any active information processing, task preparation is an active state whose role is to “bridge” resting state and information processing phases in the experiment by providing timely connection and disconnection within the WM networks.

### Limitations

One limitation of the current study is that the task performance blocks contain more data points than rest and preparation periods (>200 vs. >90 vs. 40 data points per condition). One might wonder whether the fewer data points in the analyses of the preparation periods might result in reduced power. However, based on a simulation study by Ramsey et al. (2011), we doubt that this is an issue for our study. First, Ramsey et al. point out that one of the most important factors for the identification and orientation of the connections is the number of subjects in the data set. They report a monotonic decrease in error rates with an increase in the number of subjects and indicate that, with as few as 10 subjects in a data set, identification and orientation of connections are accurate. Given that our data set included data from 16 subjects our calculations should be accurate. Second, the simulation in Ramsey et al. included longer sessions (close to the length of our n-back task blocks) and shorter sessions (close in length to our task preparation blocks). Given that the data from 10 (rather than one) simulated subjects were analyzed using the combined IMaGES and LOFS method, the accuracy of edge detection/discovery was very high (close to 100%) and quite similar for longer and shorter sessions. Orientation precision and recall were somewhat lower for shorter than for longer sessions, but the accuracy for shorter sessions was still very high (above 80%). This latter finding suggests the possibility that the accuracy of edge orientation in our study was higher for task performance than for the rest periods and task preparation.

The main hypotheses of our study were related to the correspondence of task preparation connectivity with the task performance connectivity and the connectivity at rest. Therefore, even though we identified several connections that were unique to a preparatory or a task performance condition (e.g., LFP→LIPS for the 3-back task condition, or Rprecun→RFP for the 1-back preparation condition), or that were present for some conditions, but not the others (e.g., RSFG→RIPS connection was observed for the 1-back task, 2-back preparation and task, and 3-back preparation conditions, but not for the 1-back preparation or 3-back task conditions), we did not discuss those connections. The presence of unique connections suggests that each task preparation or task performance condition may require using unique strategies of information processing. Unfortunately, the current design does not allow us to examine these possibilities. Our study also does not allow us to examine individual difference in connectivity within the Increase and Decrease networks. Each connectivity model was derived from multiple subjects. The model fit varies across subjects and may, or may not, be related to a given subject's behavioral performance. While it is possible that faster and/or more accurate performance is associated with formation of some specific connections, our study does not have enough power to test this possibility.

## Conclusion

In summary, the present study provides novel findings about the relationship between connectivity during task preparation, task performance and rest periods by demonstrating that the connectivity among the regions within the WM network is not limited to task performance. Even though preparation periods did not require any active information maintenance or manipulation, effective connectivity during task preparation did not resemble connectivity during rest periods. Instead, two connectivity sub-networks were identified. The “default” connectivity sub-network was present in all conditions including rest. We proposed that this sub-network might serve as a core system that allows one to quickly activate cognitive, perceptual and motor systems in response to the relevant stimuli. “Preparation-related” connectivity exists during task preparation and task performance, but not at rest. It is specific for each difficulty level and likely “pre-activates” cognitive resources important for performance on each specific task. One role of such “pre-activation” may be to free neural resources during task performance and to help activate and carry over the task-related rules and intentions from task preparation to task performance. Future research using graph modeling should determine whether “default” and “preparation-related” connectivity is a phenomenon generalizable to all cognitive tasks or specific to just the n-back task.

### Conflict of interest statement

The authors declare that the research was conducted in the absence of any commercial or financial relationships that could be construed as a potential conflict of interest.
